# Personal

**Published:** 1916-03

**Authors:** 


### PERSONAL.

---

Announcement of removal, travel, and other matters of interest are requested. Please report errors in the listing of any physician in the State and other directories, that we may co-operate with the State Society in securing a correct list.

---

Dr. Willis Lynn has opened an office at 91 East Avenue, Rochester, practice limited to diseases of women and children.

---

Drs. Geo. F. and Chester C. Cott of Buffalo have moved their offices to 1001 Main Street, between High and Goodrich Streets.

---

Mr. Alexander Johnson, Field Secretary of the Committee on Provision for the Feeble-Minded, gave an illustrated lecture at the Buffalo State Normal School, Feb. 7, under the joint auspices of the School and the University of Buffalo.

---

Dr. Carl E. Muench has been elected Superintendent of the Crouse-Irving Hospital of Syracuse, and Miss Marion F. Jones has been appointed Superintendent of Nurses, these appointments filling the place of the former Superintendent, Miss Mary E. Shanahan, resigned.

---

Dr. L. H. Staples of Buffalo left for a trip to Florida and Cuba Feb. 14.

---

The following changes in addresses in alumni of the Medical Dept. of the University of Buffalo have been reported: Dr. Ellen R. Spragge, 1888, from 926 Academy Street, St. Louis, to 2010 Horton Avenue, Grand Rapids; Dr. G. P. Richardson, 1876, from Middleport, N. Y. to Madison, N. J.

---

Dr. Henry Jones Mulford of Buffalo announces the removal of his office and residence to 949 Delaware Avenue. In addition to diseases of the nose, throat and ear, Dr. Mulford will take up another line of work, which may be designated

"Child Analysis." This embraces a complete physical and mental examination of the child, by means of which the grade of any child may be determined, whether he be above normal or below, and where he belongs in school or upon the playground.

---

Dr. P. H. J. Buckley, U. B. 1915, has been appointed to the house staff of the Buffalo General Hospital.

